# Supplementary material for: A Map of 3′ DNA Transduction Variants Mediated by Non-LTR Retroelements on 3202 Human Genomes
Source: Biology (Basel). 2022 Jul 8;11(7):1032. doi: 10.3390/biology11071032 (PMC9311842; doi:10.3390/biology11071032)
Supplement: Supplementary file 1 [file biology-11-01032-s001.zip › biology-1784453-supplementary/supplementary/figureS1_lengthDistributionOfTransductions.pdf]

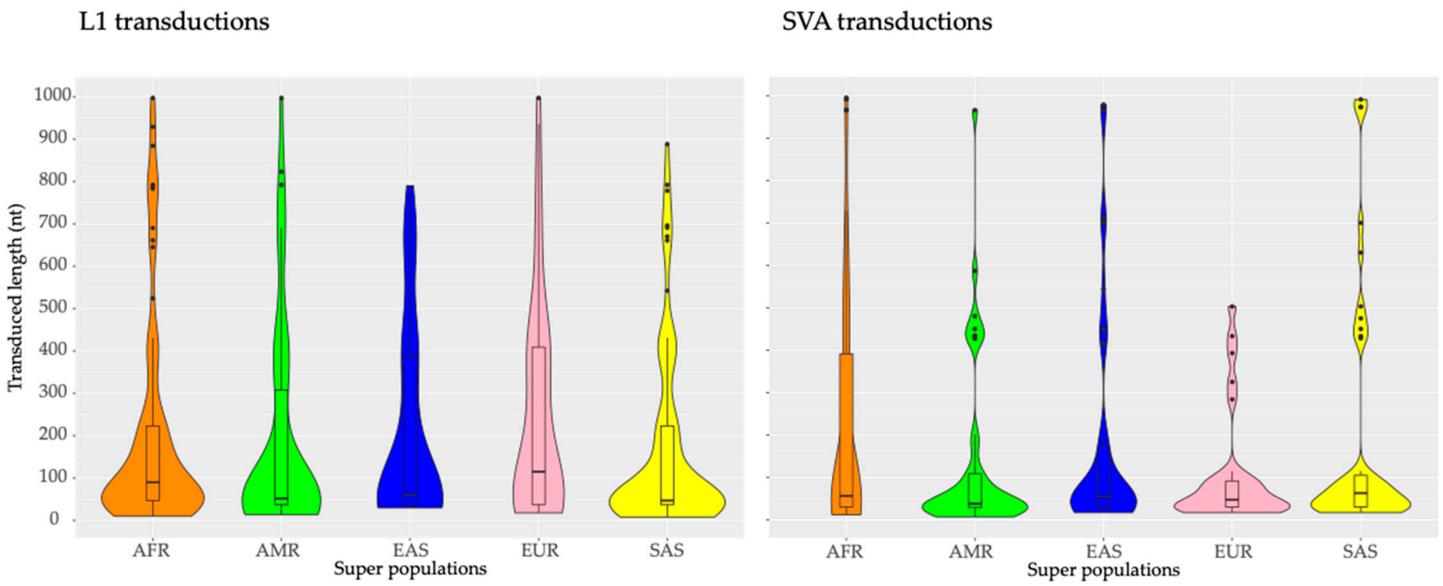

Figure S1. Length distribution of transductions mediated by L1s and SVAs across the super-populations.
