# Supplementary material for: A Map of 3′ DNA Transduction Variants Mediated by Non-LTR Retroelements on 3202 Human Genomes
Source: Biology (Basel). 2022 Jul 8;11(7):1032. doi: 10.3390/biology11071032 (PMC9311842; doi:10.3390/biology11071032)
Supplement: Supplementary file 1 [file biology-11-01032-s001.zip › biology-1784453-supplementary/supplementary/tableS1_listOfActiveRetroelementsInHumanGenome.pdf]

Table S1. Active non-LTR retroelements in the human genome based on Mills et al' s study [1]

	Subfamily
**LINE**	L1-PA2, Pre-Ta,Ta, Ta-0, Ta-1d, Ta-1nd
**SVA**	A, B, C, D, E, F

Reference

1. Mills, R. E.; Bennett, E. A.; Iskow, R. C.; Devine, S. E., Which transposable elements are active in the human genome? *Trends Genet* **2007,** *23* (4), 183-91. doi:10.1016/j.tig.2007.02.006
