# Supplementary material for: A Map of 3′ DNA Transduction Variants Mediated by Non-LTR Retroelements on 3202 Human Genomes
Source: Biology (Basel). 2022 Jul 8;11(7):1032. doi: 10.3390/biology11071032 (PMC9311842; doi:10.3390/biology11071032)
Supplement: Supplementary file 1 [file biology-11-01032-s001.zip › biology-1784453-supplementary/supplementary/tableS4_listOfSourceElementsWithTheirTransductionLength.pdf]

Table S4. Source elements of L1 and SVA transductions with their transduced length. This table summarizes the length of progenies derived from each progenitor. Numbers in parentheses are the count of each offspring with the given size.

Source locus	TE type	Transduction length in bp (count)
chr1:80939203-80945257	L1/reference	115 (1), 117 (1), 119 (1), 97 (1)
chr1:84052389-84058406	L1/reference	128 (1), 139 (1), 143 (1), 648 (1)
chr1:86679080-86685111	L1/reference	8 (1), 63 (2)
chr1:118852351-118858380	L1/reference	193 (1), 194 (1), 140 (3), 192 (3)
chr1:187343764-187349794	L1/reference	32 (1)
chr1:237019467-237025494	L1/reference	48 (1)
chr1:247687173-247693204	L1/reference	174 (1), 34 (1)
chr3:89460825-89466856	L1/reference	427 (1), 888 (1), 936 (1)
chr3:136963693-136969736	L1/reference	10 (1), 13 (1)
chr4:15841546-15847572	L1/reference	204 (1)
chr4:21159390-21165421	L1/reference	54 (1)
chr4:74717539-74723587	L1/reference	202 (1), 682 (1), 686 (1), 696 (1), 929 (1), 997 (1), 691 (3)
chr4:79966907-79972933	L1/reference	59 (1), 388 (3)
chr4:136293494-136299546	L1/reference	778 (1), 783 (1), 785 (1), 89 (1), 790 (3)
chr6:19764892-19770918	L1/reference	41 (1), 46 (1)
chr6:24811657-24817706	L1/reference	90 (2)
chr6:117102131-117108163	L1/reference	41 (1), 43 (5)
chr7:30439242-30445274	L1/reference	28 (1)
chr7:96846650-96852680	L1/reference	56 (1), 60 (2)
chr8:128453002-128459020	L1/reference	84 (1)
chr8:134070756-134076773	L1/reference	542 (1)
chr9:112798107-112804159	L1/reference	620 (1), 645 (1)
chr10:109812437-109818457	L1/reference	18 (1), 57 (1), 71 (1), 41 (2)
chr11:78677772-78683802	L1/reference	185 (1)
chr11:93136638-93142673	L1/reference	461 (1), 524 (1)

chr12:126299023-126305038	L1/reference	56 (1)
chr13:29641706-29647706	L1/reference	35 (1)
chr13:34480262-34486294	L1/reference	14 (1)
chr15:54926081-54932099	L1/reference	51 (1)
chr17:9615985-9622015	L1/reference	25 (1)
chr17:70458956-70464987	L1/reference	47 (1), 52 (1), 50 (5)
chr22:28663283-28669315	L1/reference	63 (1), 792 (1), 823 (1), 60 (4)
chrX:11707248-11713279	L1/reference	103 (1), 110 (1), 152 (1), 169 (1), 26 (1), 419 (1), 42 (1), 50 (1), 56 (1), 91 (1), 104 (2), 41 (8), 47 (10), 30 (26), 37 (35)
chrX:11935296-11941314	L1/reference	230 (1), 261 (1), 422 (1), 224 (2), 246 (2), 661 (2), 431 (4), 223 (8)
chrX:147653734-147659767	L1/reference	43 (1)
chr2_GL582966v2_alt:23969-29986	L1/reference	73 (1)
chr6_GL000253v2_alt:1206980-1213011	L1/reference	55 (1), 53 (7)
chr3:55754553	L1/non-reference	101 (1)
chr3:173031344	L1/non-reference	356 (1)
chr4:82000744	L1/non-reference	690 (1)
chr4:111707818	L1/non-reference	119 (1), 173 (1), 79 (1), 92 (1)
chr4:131260507	L1/non-reference	164 (1)
chr5:25708557	L1/non-reference	293 (1)
chr5:90154965	L1/non-reference	884 (1)
chr5:137679101	L1/non-reference	365 (1), 53 (1)
chr6:6280580	L1/non-reference	137 (1)
chr6:13190802	L1/non-reference	404 (1), 408 (1), 409 (1), 406 (2), 403 (3), 407 (3), 412 (10)
chr6:29952447	L1/non-reference	401 (1)
chr6:45358100	L1/non-reference	220 (1)
chr6:62658303	L1/non-reference	121 (1)
chr6:102398219	L1/non-reference	636 (1)
chr8:118145228	L1/non-reference	133 (1)

chr10:57219366	L1/non-reference	220 (1)
chr12:96518791	L1/non-reference	627 (1)
chr12:117376655	L1/non-reference	450 (1)
chr14:30681620	L1/non-reference	263 (1), 295 (1)
chr14:58753685	L1/non-reference	305 (1), 311 (1), 670 (4)
chr17:69181490	L1/non-reference	399 (1)
chr1:21387076-21388677	SVA/reference	991 (1)
chr1:24832277-24834863	SVA/reference	21 (1), 68 (1), 80 (1)
chr1:26817473-26818642	SVA/reference	712 (2)
chr1:27063473-27065559	SVA/reference	34 (1)
chr1:143637104-143638603	SVA/reference	979 (1)
chr2:156368891-156370126	SVA/reference	63 (2)
chr2:177946154-177947784	SVA/reference	29 (1), 55 (1)
chr2:195567221-195569191	SVA/reference	102 (1), 480 (1), 95 (2)
chr2:216225259-216227598	SVA/reference	39 (2)
chr3:48212077-48213101	SVA/reference	424 (1), 428 (1), 432 (1), 433 (1), 92 (1)
chr3:128953643-128954734	SVA/reference	991 (1)
chr3:150819421-150821828	SVA/reference	43 (1)
chr3:179120452-179121468	SVA/reference	700 (1)
chr4:1827284-1828864	SVA/reference	15 (1)
chr4:76917857-76918878	SVA/reference	72 (2)
chr4:83184624-83187035	SVA/reference	325 (1)
chr4:150658775-150661088	SVA/reference	115 (1)
chr4:183734757-183736673	SVA/reference	168 (1), 72 (1)
chr6:15189275-15190590	SVA/reference	220 (1)
chr6:27484095-27485412	SVA/reference	393 (1)
chr6:51981373-51982817	SVA/reference	81 (1), 77 (2)
chr6:56893617-56896059	SVA/reference	51 (1), 625 (1), 630 (1), 637 (1), 57 (2), 54 (3)

chr6:122847780-122849162	SVA/reference	17 (1), 30 (1), 35 (1), 77 (1), 82 (1), 76 (4)
chr7:20667753-20669743	SVA/reference	52 (1), 68 (1), 70 (1), 503 (2), 56 (5)
chr7:152584646-152586077	SVA/reference	27 (1)
chr7:155366080-155367490	SVA/reference	23 (1)
chr8:28881390-28882478	SVA/reference	356 (1)
chr8:30245944-30247879	SVA/reference	328 (1)
chr8:64685736-64687147	SVA/reference	587 (1)
chr8:128824979-128827120	SVA/reference	12 (2)
chr9:6806926-6808710	SVA/reference	17 (1)
chr9:81709536-81711924	SVA/reference	73 (1), 83 (1), 84 (1), 87 (1), 88 (1)
chr10:92376425-92377452	SVA/reference	450 (2), 449 (5)
chr10:92673727-92675979	SVA/reference	26 (1)
chr10:97276514-97277614	SVA/reference	30 (1)
chr10:99837466-99840119	SVA/reference	403 (1)
chr11:118330284-118332729	SVA/reference	33 (1)
chr11:119507011-119508903	SVA/reference	82 (1), 85 (1), 90 (1)
chr12:41300-43319	SVA/reference	18 (3)
chr12:26785353-26786469	SVA/reference	38 (1), 47 (2)
chr12:95839858-95841091	SVA/reference	43 (1), 47 (1), 60 (1)
chr14:20506451-20507630	SVA/reference	21 (1), 29 (1)
chr14:64894264-64896723	SVA/reference	140 (1), 585 (1), 590 (1)
chr14:105110790-105111988	SVA/reference	475 (1)
chr15:88551730-88554457	SVA/reference	427 (1)
chr15:89866993-89868511	SVA/reference	973 (1)
chr17:27211365-27213327	SVA/reference	44 (1), 7 (1), 31 (2)
chr17:28223104-28224648	SVA/reference	528 (1)
chr17:60550877-60551918	SVA/reference	973 (1)
chr19:29898333-29901695	SVA/reference	416 (1), 434 (1)

chr20:2824215-2825758	SVA/reference	17 (1), 544 (1), 22 (2)
chr21:17718638-17721106	SVA/reference	12 (1)
chr22:23853842-23855352	SVA/reference	995 (1), 966 (3)
chr22:40744153-40746437	SVA/reference	29 (1)
chrX:29332669-29334200	SVA/reference	40 (1)
chrX:68325837-68327795	SVA/reference	22 (2)
chrX:72680888-72681912	SVA/reference	730 (1)
chr6_GL000253v2_alt:4065841-4067131	SVA/reference	28 (1), 32 (2), 30 (19)
chr2:68079712	SVA/non-reference	181 (1)
chr6:158217807	SVA/non-reference	173 (1), 192 (1), 201 (1), 91 (1)
chr7:101347850	SVA/non-reference	284 (1)
chr9:125396733	SVA/non-reference	203 (1)
chr12:104417657	SVA/non-reference	176 (1)
